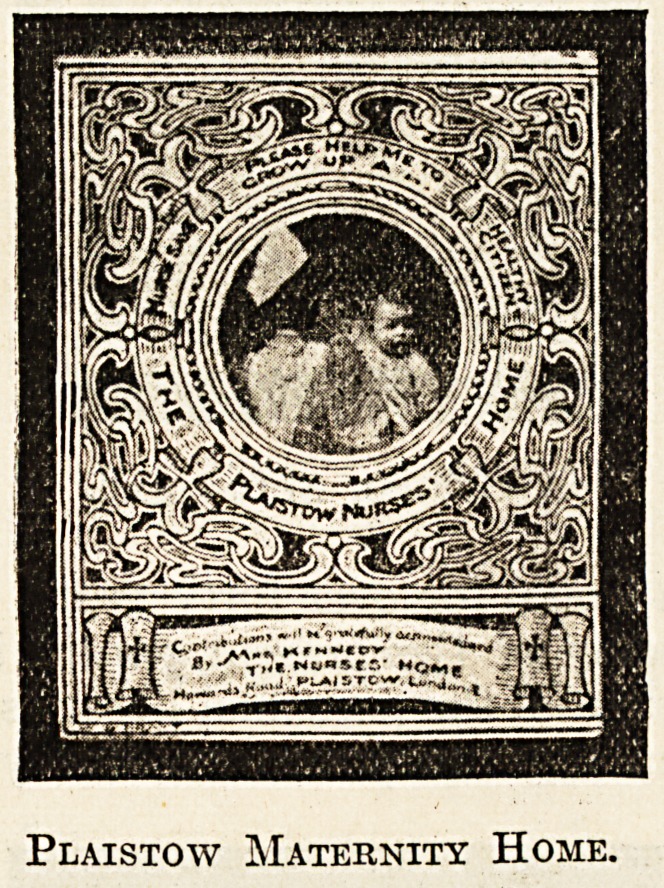# A Question for Hospital Treasurers

**Published:** 1911-09-23

**Authors:** 


### A QUESTION FOR HOSPITAL TREASURERS.

### ARE SEAL-STAMPS A POSSIBLE SOURCE OF REVENUE?

A FEW weeks ago we published a paragraph with the question-asking title, "Seal-stamps for Hospitals?" The implied question, "Should they be adopted by the voluntary hospitals" received no answer, because our readers imagined two things: first, that seal-stamps had never been obtainable, and, secondly, that no one would use them even if they were now to be had. Inadvertently, by a printer's error, we put our readers off the track by stating that a society had been formed to attempt their adoption. That sentence should have read, a society "is being formed," for whether it will succeed in passing from its embryological stage we are not in a position to say.

In the meantime, however, as August and September are the recruiting season, in which ideas germinate that they may fill the coffers of hospitals and extend the reputation of officers in the October and winter months of coming work, we publish again some further matter on this debatable topic. It is likely that any correspondence which may ensue will be instructive, and probably critical, as the subject in debate is one, at any rate, of practical interest. As all debates, however, begin with a set-piece of information, so that the coming speeches may have a solid basis from which to talk, a few brief paragraphs on the acknowledged facts in the previous history of the seal-stamp movement are appended. The question on which we would like our readers' opinions is this: "Are seal-stamps a source of hospital revenue worth attempting to exploit?" A written consensus could decide the matter. Nor is it too abstruse a question for the holidays.

One of our former Lord Mayors of London gave recently as his opinion about the seal-stamp for raising funds that, in England, nobody wanted any help in "the art of begging." At any rate, if by the more elegant and refined art of giving a fair value for money received an equal amount can be raised, without begging, it is a desirable consummation, some have said.

Seal-stamps have been sold in England hitherto in booklets of twelve to twenty stamps, of poor

THE PRESENT POPE.

artistic value and without any motto or quotation to harmonise with their design, and have left too much lacking to be either popular or vendible!

The seal-stamp has been, from its earliest initiatory use, under the highest patronage of the Court of the country that adopted it, as was the Victorian hospital-stamp of 1897. The effigies of royalty, of kings and queens, princes and prin-

A HOSPITAL STAMP IN PRUSSIA.

cesses, archdukes and archduchesses, have by high permission been printed on the seal-stamps issued for charities in the Scandinavian and Teutonic countries and Italy. There has been no stamp of

A GERMAN SEAL-STAMP.

the Vatican Court for over forty years, but a beautiful seal-stamp of the present pontiff is in use in Spain on behalf of the parochial libraries.

In Luxemburg the portrait of the young Arch-

HOSPITAL STAMP IN DENMARK.

duchess Adelaide adorns the only seal-stamp used in the Duchy for a hospital fund.

In Germany the portrait of the Empress Frederick was once executed for a hospital-stamp, but was suppressed on the decease of the lady; and the portrait of Princess Victoria with her mother was later on substituted.

The great success of the hospital seal-stamp in Denmark, Norway, and Sweden is to be attributed to the favour of the Post Office Department, which, unlike Mr. Herbert Samuel's department in London, facilitates the sale of hospital seal-stamps, the Post Office officials receiving 10 per cent. from the sales as a return for the extra labour involved.

During December every year since 1904, when Postmaster Holböll introduced this system into

Canada's Hospital Stamp.

Denmark, a Yuletide hospital-stamp has been printed for Denmark and for the Danish West Indies. Norway and Sweden now also issue a Yuletide stamp, and accord to the Post Office officials 10 per cent. on the annual sales. In Sweden, one Christmas, £50,000 was the amount realised.

Belgium and Portugal also favour the issue of hospital-stamps, but the stamps that have appeared are poor and commonplace in execution and have

Plaistow Maternity Home.

no word beyond "Caritas" to recommend their scope or intention.

Despite the thousands of various seal-stamps in vogue on the Continent, very few are of a nature worthy to be called a work of art. The Berlin illustrated paper "Die Woche" ("The Week"), in March 1908 offered a competition prize of 3,000 Marks for the best design for a charity seal-stamp, and in July of the same year published the result. The first prize of 1,000 Marks was awarded to Mr. Ad. Möller, of Altona, on the Elbe, for a ship with the canvas well stretched and a cross on the mainsail. The design is very simple. Perhaps the draughtsman had in his mind the English expression "raising the wind."

The result of the competition was absolutely null as to any expression of art applied to charity.

British Hospitals and Using Them.

In the United States of North America the "Christmas hospital-stamp" is in some favour. It is used also for the Canada Muskoka Free Hospital for Consumptives, under Lord Strathcona's patronage. The recent stamp adopted by Lord Mayor Treloar for the Alton Cripple Home is not the first attempt to apply the seal-stamp to a charity in London, for the Maternity Charity and Nurses' Home, Plaistow, has also its humble seal-stamp, but neither of these stamps is artistic or likely to sell except because of the object for which they have been issued; whereas a seal-stamp should sell on its own merits and be popular and please the purchaser for its intrinsic value first of all.

## Figures and Tables

**Figure f1:**
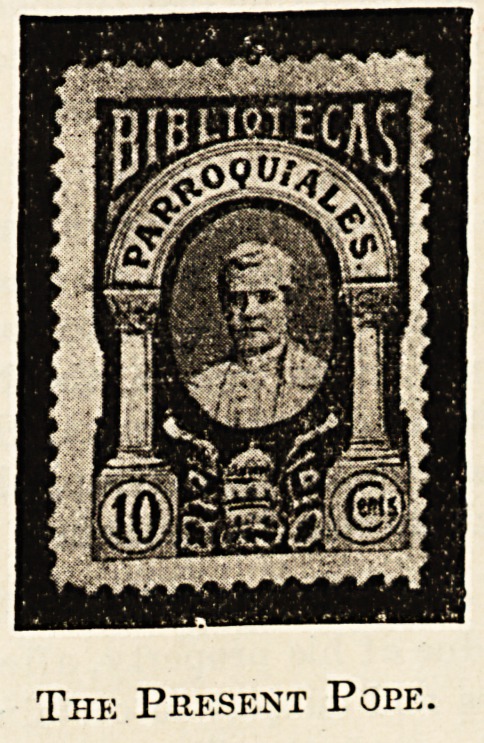


**Figure f2:**
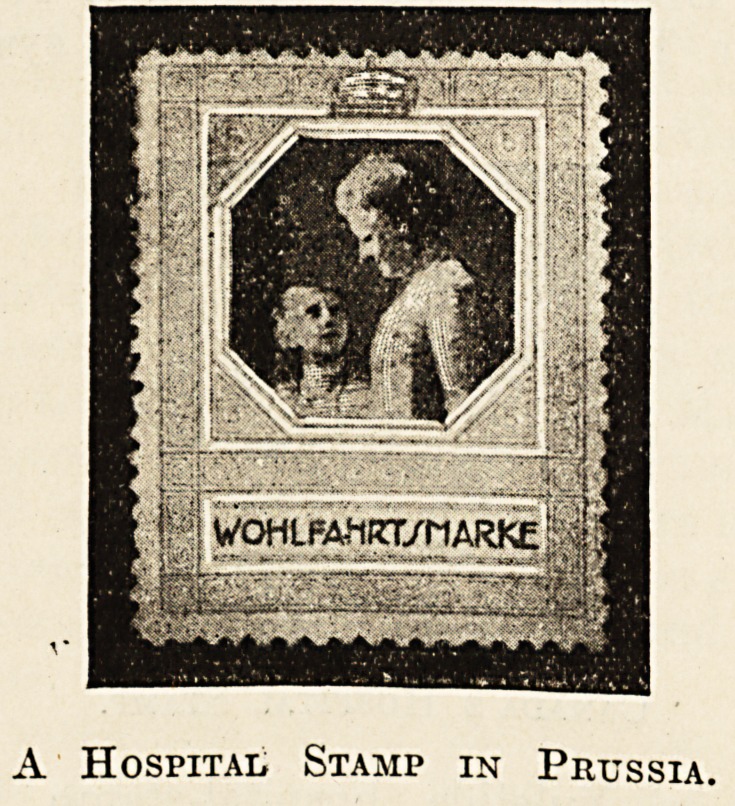


**Figure f3:**
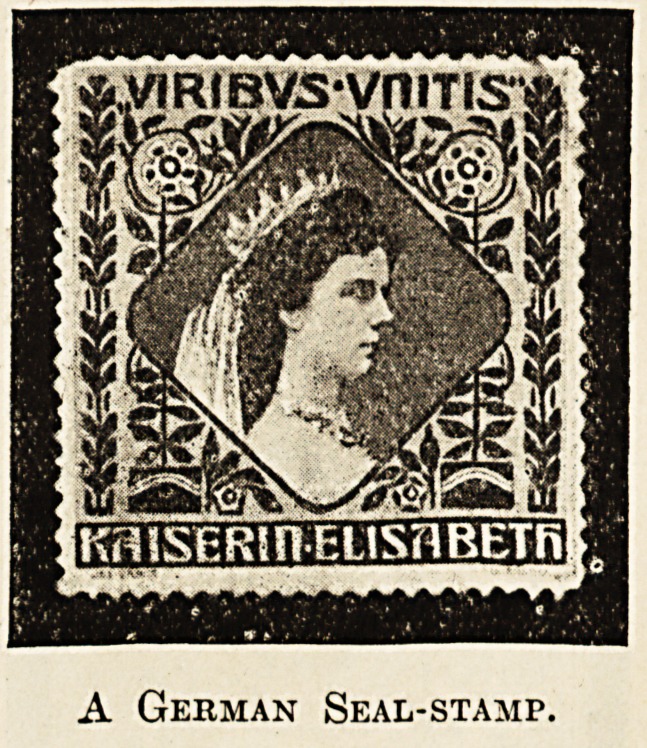


**Figure f4:**
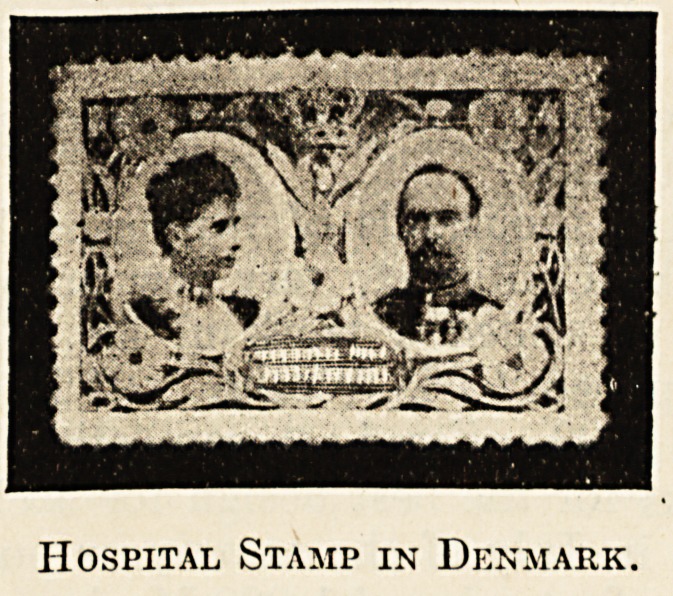


**Figure f5:**
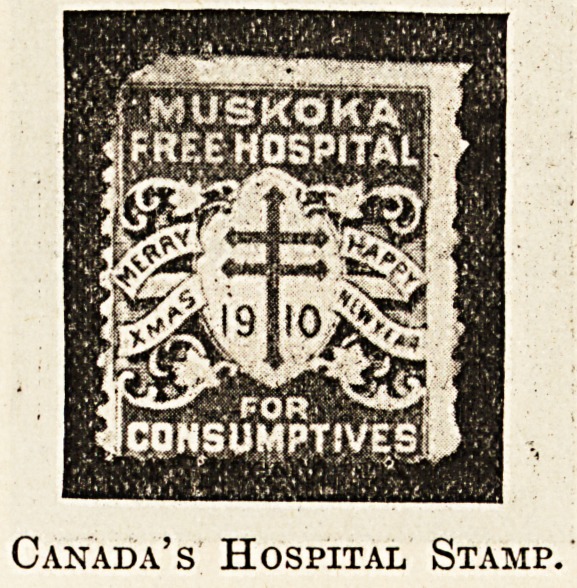


**Figure f6:**